# An accurate approximation formula for gamma function

**DOI:** 10.1186/s13660-018-1646-6

**Published:** 2018-03-06

**Authors:** Zhen-Hang Yang, Jing-Feng Tian

**Affiliations:** 1 College of Science and Technology, North China Electric Power University, Baoding, P.R. China; 2 Department of Science and Technology, State Grid Zhejiang Electric Power Company Research Institute, Hangzhou, China

**Keywords:** 33B15, 26D15, 26A48, 26A51, Gamma function, Monotonicity, Convexity, Approximation

## Abstract

In this paper, we present a very accurate approximation for the gamma function:

$$ \Gamma( x+1 ) \thicksim\sqrt{2\pi x} \biggl( \frac{x}{e} \biggr) ^{x} \biggl( x\sinh\frac{1}{x} \biggr) ^{x/2}\exp \biggl( \frac{7}{324}\frac{1}{x^{3} ( 35x^{2}+33 ) } \biggr) =W_{2} ( x ) $$

as $x\rightarrow\infty$, and we prove that the function $x\mapsto\ln \Gamma ( x+1 ) -\ln W_{2} ( x ) $ is strictly decreasing and convex from $( 1,\infty ) $ onto $( 0,\beta ) $, where

$$ \beta=\frac{22{,}025}{22{,}032}-\ln\sqrt{2\pi\sinh1}\approx0.00002407. $$

## Introduction

The Stirling formula states that

1.1 $$ n!\thicksim\sqrt{2\pi n}n^{n}e^{-n} $$

for $n\in\mathbb{N}$. The gamma function $\Gamma ( x ) =\int_{0}^{\infty}t^{x-1}e^{-t}\,dt$ for $x>0$ is a generalization of the factorial function *n*! and has important applications in various branches of mathematics; see, for example, [1–6] and the references cited therein.

There are many refinements for the Stirling formula; see, for example, Burnside’s [7], Gosper [8], Batir [9], Mortici [10]. Many authors pay attention to find various better approximations for the gamma function, for instance, Ramanujan [11, P. 339], Smith [12, Eq. (42)], [13], Mortici [14], Nemes [15, Corollary 4.1], Yang and Chu [16, Propositions 4 and 5], Chen [17].

More results involving the approximation formulas for the factorial or gamma function can be found in [16, 18–27] and the references cited therein. Several nice inequalities between gamma function and the truncations of its asymptotic series can be found in [28, 29].

Now let us focus on the Windschitl approximation formula (see [12, Eq. (42)], [13]) defined by

1.2 $$ \Gamma( x+1 ) \thicksim\sqrt{2\pi x} \biggl( \frac{x}{e} \biggr) ^{x} \biggl( x\sinh\frac{1}{x} \biggr) ^{x/2}:=W_{0} ( x )\quad \text{as }x\rightarrow\infty. $$

As shown in [17], the rate of Windschitl’s approximation $W_{0} ( x ) $ converging to $\Gamma ( x+1 ) $ is like $x^{-5}$ as $x\rightarrow\infty$, and it is faster on replacing $W_{0} ( x ) $ by

1.3 $$ W_{1} ( x ) =\sqrt{2\pi x} \biggl( \frac{x}{e} \biggr) ^{x} \biggl( x\sinh\frac{1}{x}+\frac{1}{810x^{6}} \biggr) ^{x/2} $$

(see [13]). These results show that $W_{0} ( x ) $ and $W_{1} ( x ) $ are excellent approximations for the gamma function.

In 2009, Alzer [30] proved that, for all $x>0$,

1.4 $$\begin{aligned} &\sqrt{2\pi x} \biggl( \frac{x}{e} \biggr) ^{x} \biggl( x\sinh \frac{1}{x} \biggr) ^{x/2} \biggl( 1+\frac{\alpha}{x^{5}} \biggr) \\ &\quad< \Gamma( x+1 ) =\sqrt{2\pi x} \biggl( \frac{x}{e} \biggr) ^{x} \biggl( x\sinh\frac{1}{x} \biggr) ^{x/2} \biggl( 1+ \frac{\beta}{x^{5}} \biggr) \end{aligned}$$

with the best possible constants $\alpha=0$ and $\beta=1/1620$. Lu, Song and Ma [31] extended Windschitl’s formula to

$$ \Gamma( n+1 ) \thicksim\sqrt{2\pi n} \biggl( \frac{n}{e} \biggr) ^{n} \biggl[ n\sinh\biggl( \frac{1}{n}+\frac{a_{7}}{n^{7}}+ \frac{a_{9}}{n^{9}}+\frac{a_{11}}{n^{11}}+\cdots\biggr) \biggr] ^{n/2} $$

with $a_{7}=1/810,a_{9}=-67/42{,}525,a_{11}=19/8505,\ldots $ . An explicit formula for determining the coefficients of $n^{-k}$ ($n\in\mathbb{N}$) was given in [32, Theorem 1] by Chen. Another asymptotic expansion

1.5 $$ \Gamma( x+1 ) \thicksim\sqrt{2\pi x} \biggl( \frac{x}{e} \biggr) ^{x} \biggl( x\sinh\frac{1}{x} \biggr) ^{x/2+\sum_{j=0}^{\infty }r_{j}x^{-j}}, \quad x\rightarrow\infty $$

was presented in the same reference [32, Theorem 2].

Motivated by the above comments, the aim of this paper is to provide a more accurate Windschitl type approximation:

1.6 $$ \Gamma( x+1 ) \thicksim\sqrt{2\pi x} \biggl( \frac{x}{e} \biggr) ^{x} \biggl( x\sinh\frac{1}{x} \biggr) ^{x/2}\exp \biggl( \frac{7}{324}\frac{1}{x^{3} ( 35x^{2}+33 ) } \biggr) =W_{2} ( x ) $$

as $x\rightarrow\infty$. Our main result is the following theorem.

### Theorem 1

*The function*

$$ f_{0} ( x ) =\ln\Gamma( x+1 ) -\ln\sqrt{2\pi}- \biggl( x+ \frac{1}{2} \biggr) \ln x+x-\frac{x}{2}\ln\biggl( x\sinh \frac{1}{x} \biggr) -\frac{7}{324}\frac{1}{x^{3} ( 35x^{2}+33 ) } $$

*is strictly decreasing and convex from* $( 1,\infty ) $ *onto* $( 0,f_{0} ( 1 ) ) $, *where*

$$ f_{0} ( 1 ) =\frac{22{,}025}{22{,}032}-\ln\sqrt{2\pi\sinh1}\approx 0.00002407. $$

## Lemmas

An important research subject in analyzing inequality is to convert an univariate into the monotonicity of functions [33–35]. Since the function $f_{0} ( x ) $ contains gamma and hyperbolic functions, it is very hard to deal with its monotonicity and convexity by usual approaches. For this purpose, we need the following lemmas, which provide a new way to prove our result.

### Lemma 1

*The inequality*

$$ \psi^{\prime} \biggl( x+\frac{1}{2} \biggr) >x\frac{x^{4}+\frac {227}{66}x^{2}+\frac{4237}{2640}}{x^{6}+\frac{155}{44}x^{4}+\frac{329}{176}x^{2}+\frac {375}{4928}} $$

*holds for* $x>0$.

### Proof

Let

$$ g_{1} ( x ) =\psi^{\prime} \biggl( x+\frac{1}{2} \biggr) -x\frac{x^{4}+\frac{227}{66}x^{2}+\frac{4237}{2640}}{x^{6}+\frac {155}{44}x^{4}+\frac{329}{176}x^{2}+\frac{375}{4928}}. $$

Then by the recurrence formula [36, p. 260, (6.4.6)]

$$ \psi^{\prime} ( x+1 ) -\psi^{\prime} ( x ) =-\frac{1}{x^{2}} $$

we have

$$\begin{aligned} &g_{1} ( x+1 ) -g_{1} ( x ) \\ &\quad=\psi^{\prime} \biggl( x+\frac{3}{2} \biggr) -\frac{ ( x+1 ) ( ( x+1 ) ^{4}+\frac{227}{66} ( x+1 ) ^{2}+\frac{4237}{2640} ) }{ ( x+1 ) ^{6}+\frac{155}{44} ( x+1 ) ^{4}+\frac{329}{176} ( x+1 ) ^{2}+\frac{375}{4928}} \\ &\qquad{}-\psi^{\prime} \biggl( x+\frac{1}{2} \biggr) +\frac{x ( x^{4}+\frac {227}{66}x^{2}+\frac{4237}{2640} ) }{x^{6}+\frac{155}{44}x^{4}+\frac{329}{176}x^{2}+\frac{375}{4928}} \\ &\quad =-58{,}982{,}400 ( 2x+1 ) ^{-2} \bigl( 4928x^{6}+17{,}360x^{4}+9212x^{2}+375 \bigr) ^{-1} \\ &\qquad{}\times \bigl( 4928x^{6}+29{,}568x^{5}+91{,}280x^{4}+168{,}000x^{3}+187{,}292x^{2}\\ &\qquad{}+117{,}432x+31 {,}875 \bigr) ^{-1}\\ &\quad< 0. \end{aligned}$$

It then follows that

$$ g_{1} ( x ) >g_{1} ( x+1 ) >\cdots>\lim_{n\rightarrow\infty }g_{1} ( x+n ) =0, $$

which proves the desired inequality, and the proof is done. □

### Lemma 2

*The inequalities*

2.1 $$ \frac{t}{\sinh t}>1-\frac{1}{6}t^{2}+\frac{7}{360}t^{4}- \frac{31}{15{,}120}t^{6}+\frac{127}{604{,}800}t^{8}- \frac{73}{3{,}421{,}440}t^{10}>0 $$

*hold for* $t\in(0,1]$.

### Proof

It was proved in [29, Theorem 1] that, for integer $n\geq0$, the double inequality

2.2 $$ -\sum_{i=0}^{2n+1}\frac{2 ( 2^{2i-1}-1 ) B_{2i}}{ ( 2i ) !}t^{2i-1}< \frac{1}{\sinh t}< -\sum_{i=0}^{2n} \frac{2 ( 2^{2i-1}-1 ) B_{2i}}{ ( 2i ) !}t^{2i-1} $$

holds for $x>0$. Taking $n=2$ yields

$$ \frac{1}{\sinh t}>\frac{1}{t}-\frac{1}{6}t+\frac{7}{360}t^{3}- \frac{31}{15{,}120}t^{5}+\frac{127}{604{,}800}t^{7}- \frac{73}{3{,}421{,}440}t^{9}:=\frac{h ( t ) }{t}, $$

which is equivalent to the first inequality of (2.1) for all $t>0$.

Since $x\in(0,1]$, making a change of variable $t^{2}=1-x\in(0,1]$ we obtain

$$\begin{aligned} h ( t ) ={}&\frac{73}{3{,}421{,}440}x^{5}+\frac{12{,}371}{119{,}750{,}400}x^{4}+ \frac{85{,}243}{59{,}875{,}200}x^{3} \\ &{}+\frac{858{,}623}{59{,}875{,}200}x^{2}+\frac {15{,}950{,}191}{119{,}750{,}400}x+\frac{14{,}556{,}793}{17{,}107{,}200}>0, \end{aligned}$$

which proves the second one, and the proof is complete. □

The following lemma offers a simple criterion to determine the sign of a class of special polynomial on given interval contained in $( 0,\infty ) $ without using Descartes’ rule of signs, which play an important role in studying certain special functions; see for example [37, 38]. A series version can be found in [39].

### Lemma 3

([37, Lemma 7])

*Let* $n\in\mathbb{N}$ *and* $m\in\mathbb{N}\cup\{0\}$ *with* $n>m$ *and let* $P_{n} ( t ) $ *be a polynomial of degree* *n* *defined by*

2.3 $$ P_{n} ( t ) =\sum_{i=m+1}^{n}a_{i}t^{i}- \sum_{i=0}^{m}a_{i}t^{i}, $$

*where* $a_{n},a_{m}>0$, $a_{i}\geq0$ *for* $0\leq i\leq n-1$ *with* $i\neq m$. *Then there is a unique number* $t_{m+1}\in ( 0,\infty ) $ *satisfying* $P_{n} ( t ) =0$ *such that* $P_{n} ( t ) <0$ *for* $t\in ( 0,t_{m+1} ) $ *and* $P_{n} ( t ) >0$ *for* $t\in ( t_{m+1},\infty ) $.

*Consequently*, *for given* $t_{0}>0$, *if* $P_{n} ( t_{0} ) >0$ *then* $P_{n} ( t ) >0$ *for* $t\in ( t_{0},\infty ) $ *and if* $P_{n} ( t_{0} ) <0$ *then* $P_{n} ( t ) <0$ *for* $t\in ( 0,t_{0} ) $.

## Proof of Theorem 1

With the aid of the lemmas in Sect. 2, we can prove Theorem 1.

### Proof of Theorem 1

Differentiation yields

$$\begin{aligned} &f_{0}^{\prime} ( x ) =\psi( x+1 ) -\frac{1}{2}\ln \biggl( x\sinh\frac{1}{x} \biggr) +\frac{1}{2x}\coth \frac{1}{x} \\ &\phantom{f_{0}^{\prime} ( x )=}{}-\ln x-\frac{1}{2x}-\frac{1}{2}+\frac{7}{324} \frac{175x^{2}+99}{x^{4} ( 35x^{2}+33 ) ^{2}}, \\ &f_{0}^{\prime\prime} ( x ) =\psi^{\prime} ( x+1 ) +\frac{1}{2x^{3}}\frac{1}{\sinh^{2} ( 1/x ) } \\ &\phantom{f_{0}^{\prime\prime} ( x ) =}{}-\frac{3}{2x}+\frac{1}{2x^{2}}-\frac{7}{54}\frac {6125x^{4}+6545x^{2}+2178}{x^{5} ( 35x^{2}+33 ) ^{3}}. \end{aligned}$$

Since $\lim_{x\rightarrow\infty}f_{0} ( x ) =\lim_{x\rightarrow \infty}f_{0}^{\prime} ( x ) =0$, it suffices to prove $f_{0}^{\prime\prime} ( x ) >0$ for $x\geq1$. Replacing *x* by $( x+1/2 ) $ in Lemma 1 leads to

$$ \psi^{\prime} ( x+1 ) >\frac{7}{30}\frac{ ( 2x+1 ) ( 165x^{4}+330x^{3}+815x^{2}+650x+417 ) }{77x^{6}+231x^{5}+560x^{4}+735x^{3}+623x^{2}+294x+60}, $$

which indicates that

$$\begin{aligned} f_{0}^{\prime\prime} ( x ) >{}&\frac{7}{30}\frac{ ( 2x+1 ) ( 165x^{4}+330x^{3}+815x^{2}+650x+417 ) }{77x^{6}+231x^{5}+560x^{4}+735x^{3}+623x^{2}+294x+60}+ \frac{1}{2x^{3}}\frac{1}{\sinh^{2} ( 1/x ) } \\ &{}-\frac{3}{2x}+\frac{1}{2x^{2}}-\frac{7}{54}\frac {6125x^{4}+6545x^{2}+2178}{x^{5} ( 35x^{2}+33 ) ^{3}}:=f_{01} \biggl( \frac{1}{x} \biggr). \end{aligned}$$

Arranging gives

$$\begin{aligned} f_{01} ( t ) ={}&\frac{t}{2} \biggl( \frac{t}{\sinh t} \biggr) ^{2}+\frac{7}{30}\frac{t ( t+2 ) ( 417t^{4}+650t^{3}+815t^{2}+330t+165 ) }{60t^{6}+294t^{5}+623t^{4}+735t^{3}+560t^{2}+231t+77} \\ &{}-\frac{3}{2}t+\frac{1}{2}t^{2}-\frac{7}{54}t^{7} \frac{2178t^{4}+6545t^{2}+6125}{ ( 33t^{2}+35 ) ^{3}}, \end{aligned}$$

where $t=1/x\in ( 0,1 ) $. Applying the first inequality of (2.1) we have

$$\begin{aligned} f_{01} ( t ) >{}&\frac{t}{2} \biggl( 1-\frac{1}{6}t^{2}+ \frac{7}{360}t^{4}-\frac{31}{15{,}120}t^{6}+ \frac{127}{604{,}800}t^{8}-\frac{73}{3{,}421{,}440}t^{10} \biggr) ^{2} \\ &{}+\frac{7}{30}\frac{t ( t+2 ) ( 417t^{4}+650t^{3}+815t^{2}+330t+165 ) }{60t^{6}+294t^{5}+623t^{4}+735t^{3}+560t^{2}+231t+77} \\ &{}-\frac{3}{2}t+\frac{1}{2}t^{2}-\frac{7}{54}t^{7} \frac{2178t^{4}+6545t^{2}+6125}{ ( 33t^{2}+35 ) ^{3}} \\ ={}&\frac{t^{11}\times p_{22} ( t ) }{ ( 33t^{2}+35 ) ^{3} ( 60t^{6}+294t^{5}+623t^{4}+735t^{3}+560t^{2}+231t+77 ) }, \end{aligned}$$

where $p_{22} ( t ) =\sum_{k=0}^{22}a_{k}t^{k}$ with $a_{0}=\frac{2{,}341{,}955}{27}$, $a_{1}=\frac{2{,}341{,}955}{9}$, $a_{2}= \frac{4{,}592{,}761{,}525{,}177}{41{,}057{,}280}$, $a_{3}= \frac {3{,}740{,}791{,}861{,}177}{13{,}685{,}760}$, $a_{4}= -\frac{21{,}774{,}907{,}040{,}747}{615{,}859{,}200}$, $a_{5}=\frac {1{,}776{,}198{,}096{,}757}{51{,}321{,}600}$, $a_{6}=-\frac{2{,}348{,}474{,}362{,}865{,}491}{59{,}122{,}483{,}200} $, $a_{7}=-\frac{444{,}392{,}576{,}792{,}851}{19{,}707{,}494{,}400}$, $a_{8}= \frac {722{,}576{,}509{,}559{,}549}{344{,}881{,}152{,}000} $, $a_{9}=\frac {734{,}284{,}235{,}570{,}623}{229{,}920{,}768{,}000}$, $a_{10}=-\frac {27{,}685{,}269{,}148{,}007{,}477}{74{,}494{,}328{,}832{,}000}$, $a_{11}=-\frac {13{,}202{,}571{,}814{,}150{,}457}{24{,}831{,}442{,}944{,}000}$, $a_{12}=\frac{1{,}859{,}898{,}503{,}651{,}431}{585{,}312{,}583{,}680{,}000}$, $a_{13}=\frac{40{,}990{,}762{,}057{,}313{,}921}{682{,}864{,}680{,}960{,}000}$, $a_{14}=\frac{1{,}227{,}464{,}630{,}525{,}327}{573{,}606{,}332{,}006{,}400}$, $a_{15}=-\frac{107{,}829{,}513{,}340{,}517}{19{,}510{,}419{,}456{,}000}$, $a_{16}=-\frac{1{,}469{,}516{,}232{,}022{,}339}{4{,}780{,}052{,}766{,}720{,}000}$, $a_{17}=\frac{224{,}320{,}158{,}179}{492{,}687{,}360{,}000}$, $a_{18}=\frac{214{,}165{,}238{,}137}{6{,}437{,}781{,}504{,}000}$, $a_{19}=-\frac{402{,}182{,}039}{11{,}943{,}936{,}000}$, $a_{20}=-\frac{150{,}639{,}953}{50{,}164{,}531{,}200}$, $a_{21}= \frac{2{,}872{,}331}{1{,}194{,}393{,}600}$, $a_{22}=\frac{58{,}619}{119{,}439{,}360}$.

It remains to prove $p_{22} ( t ) =\sum_{k=0}^{22}a_{k}t^{k}>0$ for $t\in(0,1]$. Since $a_{k}>0$ for $k=0$, 1, 2, 3, 8, 9, 12, 13, 14, 17, 18, 21, 22 and $a_{k}<0$ for $k=4$, 6, 7, 10, 11, 15, 16, 19, 20, we have

$$ p_{22} ( t ) =\sum_{k=0}^{22}a_{k}t^{k}= \sum_{a_{k}>0}a_{k}t^{k}+\sum_{a_{k}< 0}a_{k}t^{k}>\sum _{k=4,6,7,10,11,15,16,19,20}a_{k}t^{k}+\sum _{k=0}^{3}a_{k}t^{k}:=p_{20} ( t ). $$

Clearly, the coefficients of the polynomial $-p_{20} ( t ) $ satisfy the conditions in Lemma 3, and

$$ -p_{20} ( 1 ) =\sum_{k=4,6,7,10,11,15,16,19,20} ( -a_{k} ) -\sum_{k=0}^{3}a_{k}=- \frac{1{,}135{,}768{,}202{,}621{,}781{,}774{,}901}{1{,}792{,}519{,}787{,}520{,}000}< 0. $$

It then follows that $p_{20} ( t ) >0$ for $t\in(0,1]$, and so is $p_{22} ( t ) $, which implies $f_{01} ( t ) >0$ for $t\in (0,1]$. Consequently, $f_{0}^{\prime\prime} ( x ) >0$ for all $x\geq1$. This completes the proof. □

As a direct consequence of Theorem 1, we immediately get the following.

### Corollary 1

*For* $n\in\mathbb{N}$, *the double inequality*

$$ \exp\frac{7}{324n^{3} ( 35n^{2}+33 ) }< \frac{n!}{\sqrt{2\pi n}( n/e ) ^{n} ( n\sinh n^{-1} ) ^{n/2}}< \lambda\exp\frac{7}{324n^{3} ( 35n^{2}+33 ) } $$

*holds with the best constant*

$$ \lambda=\exp f_{0} ( 1 ) =\frac{1}{\sqrt{2\pi\sinh1}}\exp\frac{22{,}025}{22{,}032} \approx1.000024067. $$

Set

$$ D_{0} ( y ) =y-\ln( 1+y ),\quad y=\frac{7}{324x^{3} ( 35x^{2}+33 ) }. $$

Then it is easy to check that, for $x>1$,

$$\begin{aligned} &\frac{dD_{0} ( y ) }{dx}=-\frac{49}{324}\frac{175x^{2}+99}{x^{4} ( 35x^{2}+33 ) ^{2} ( 11{,}340x^{5}+10{,}692x^{3}+7 ) }< 0, \\ &\frac{d^{2}D_{0} ( y ) }{dx^{2}}=\frac{343}{54}\frac{ ( 18{,}191{,}250x^{9}+37{,}110{,}150x^{7}+24{,}992{,}550x^{5}+6125x^{4}+5{,}821 {,}794x^{3}+6545x^{2}+2178 ) }{x^{5} ( 35x^{2}+33 ) ^{3} ( 11{,}340x^{5}+10{,}692x^{3}+7 ) ^{2}}\\ &\phantom{\frac{d^{2}D_{0} ( y ) }{dx^{2}}}>0. \end{aligned}$$

That is to say, $x\mapsto D_{0} ( y ) $ is decreasing and convex on $( 1,\infty ) $, and so is the function $f_{0}^{\ast} ( x ):=f_{0} ( x ) +D_{0} ( y ) $ by Theorem 1.

### Corollary 2

*The function*

$$\begin{aligned} f_{0}^{\ast} ( x ) ={}&\ln\Gamma( x+1 ) -\ln\sqrt{2\pi }- \biggl( x+\frac{1}{2} \biggr) \ln x+x-\frac{x}{2}\ln \biggl( x\sinh\frac{1}{x}\biggr) \\ &{}-\ln\biggl( 1+ \frac{7}{324x^{3} ( 35x^{2}+33 ) } \biggr) \end{aligned}$$

*is strictly decreasing and convex from* $( 1,\infty ) $ *onto* $( 0,f_{0}^{\ast} ( 1 ) ) $, *where*

$$ f_{0}^{\ast} ( 1 ) =1-\ln\frac{22{,}039}{22{,}032}-\ln\sqrt{2\pi \sinh1}\approx0.00002412. $$

### Remark 1

Corollary 2 offers another approximation formula

3.1 $$ \Gamma( x+1 ) \thicksim\sqrt{2\pi x} \biggl( \frac{x}{e} \biggr) ^{x} \biggl( x\sinh\frac{1}{x} \biggr) ^{x/2} \biggl( 1+ \frac{7}{324}\frac{1}{x^{3} ( 35x^{2}+33 ) } \biggr) =W_{2}^{\ast} ( x ) . $$

Also, for $n\in\mathbb{N}$,

$$\begin{aligned} 1+\frac{7}{324n^{3} ( 35n^{2}+33 ) }< \frac{n!}{\sqrt{2\pi n} ( n/e ) ^{n} ( n\sinh n^{-1} ) ^{n/2}}< \lambda^{\ast} \biggl( 1+\frac{7}{324n^{3} ( 35n^{2}+33 ) } \biggr) \end{aligned}$$

with the best constant

$$ \lambda^{\ast}=\exp f_{0}^{\ast} ( 1 ) = \frac{22{,}032}{22{,}039}\frac{e}{\sqrt{2\pi\sinh1}}\approx1.000024117. $$

## Numerical comparisons

It is well known that an excellent approximation for the gamma function is fairly accurate but relatively simple. In this section, we list some known approximation formulas for the gamma function and compare them with $W_{1} ( x ) $ given by (1.3) and our new one $W_{2} ( x ) $ defined by (1.6).

It has been shown in [17] that, as $x\rightarrow \infty$, Ramanujan’s [11, P. 339] approximation formula holds,

$$ \Gamma( x+1 ) \thicksim\sqrt{\pi} \biggl( \frac{x}{e} \biggr) ^{x} \biggl( 8x^{3}+4x^{2}+x+\frac{1}{30} \biggr) ^{1/6} \biggl( 1+O \biggl( \frac{1}{x^{4}} \biggr) \biggr) :=R ( x ), $$

and Smith’s one [12, Eq. (42)],

$$ \Gamma\biggl( x+\frac{1}{2} \biggr) \thicksim\sqrt{2\pi} \biggl( \frac{x}{e}\biggr) ^{x} \biggl( 2x\tanh \frac{1}{2x} \biggr) ^{x/2} \biggl( 1+O \biggl( \frac{1}{x^{5}} \biggr) \biggr):=S ( x ), $$

Nemes’ one [15, Corollary 4.1],

$$ \Gamma( x+1 ) \thicksim\sqrt{2\pi x} \biggl( \frac{x}{e} \biggr) ^{x} \biggl( 1+\frac{1}{12x^{2}-1/10} \biggr) ^{x} \biggl( 1+O \biggl( \frac{1}{x^{5}} \biggr) \biggr) =:N_{1} ( x ), $$

and Chen’s one [17],

4.1 $$\begin{aligned} \Gamma(x+1)&\thicksim\sqrt{2\pi x} \biggl( \frac{x}{e} \biggr) ^{x} \biggl( 1+\frac{1}{12x^{3}+24x/7-1/2} \biggr) ^{x^{2}+53/210} \biggl( 1+O \biggl( \frac{1}{x^{7}} \biggr) \biggr) \\ &:=C ( x ). \end{aligned}$$

Moreover, it is easy to check that Nemes’ result [13] is another one,

4.2 $$\begin{aligned} \Gamma( x+1 ) \thicksim\sqrt{2\pi x} \biggl( \frac{x}{e} \biggr) ^{x}\exp\biggl( \frac{210x^{2}+53}{360x ( 7x^{2}+2 ) } \biggr) \biggl( 1+O \biggl( \frac{1}{x^{7}} \biggr) \biggr):=N_{2} ( x ), \end{aligned}$$

and so are Yang and Chu’s [16, Propositions 4 and 5] ones,

$$\begin{aligned} &\Gamma\biggl( x+\frac{1}{2} \biggr) =\sqrt{2\pi} \biggl( \frac{x}{e}\biggr) ^{x}\exp\biggl( - \frac{1}{24}\frac{x}{x^{2}+7/120} \biggr) \biggl( 1+O \biggl( \frac{1}{x^{5}} \biggr) \biggr):=Y_{1} ( x ), \\ &\Gamma\biggl( x+\frac{1}{2} \biggr) =\sqrt{2\pi} \biggl( \frac{x}{e}\biggr) ^{x}\exp\biggl( - \frac{1}{24x}+\frac{7}{2880x}\frac{1}{x^{2}+31/98}\biggr) \biggl( 1+O \biggl( \frac{1}{x^{7}} \biggr) \biggr):=Y_{2} ( x ), \end{aligned}$$

and we have Windschitl one [13],

$$\begin{aligned} \Gamma(x+1)\thicksim\sqrt{2\pi x} \biggl( \frac{x}{e} \biggr) ^{x} \biggl( x\sinh\frac{1}{x}+\frac{1}{810x^{6}} \biggr) ^{x/2} \biggl( 1+O \biggl( \frac{1}{x^{7}} \biggr) \biggr) =W_{1} ( x ). \end{aligned}$$

For our new ones $W_{2} ( x ) $ given in (1.6) and its counterpart $W_{2}^{\ast} ( x ) $ given in (3.1), we easily check that

$$ \lim_{x\rightarrow\infty}\frac{\ln\Gamma ( x+1 ) -\ln W_{2} ( x ) }{x^{-9}}=\lim_{x\rightarrow\infty} \frac{\ln\Gamma ( x+1 ) -\ln W_{2}^{\ast} ( x ) }{x^{-9}}=\frac{869}{2{,}976{,}750}, $$

which show that the rates of $W_{2} ( x ) $ and $W_{2}^{\ast } ( x ) $ converging to $\Gamma ( x+1 ) $ are both as $x^{-9}$.

From these, we see that our new Windschitl type approximation formulas $W_{2} ( x ) $ and $W_{2}^{\ast} ( x ) $ are best among those listed above, which can also be seen from Table 1.

**Table 1:** Comparison among $N_{2}$ (4.2), *C* (4.1), $W_{1}$ (1.3) and $W_{2}$ (1.6)

*x*	$\vert \frac{N_{2} ( x ) -\Gamma ( x+1 ) }{\Gamma ( x+1 ) } \vert $	$\vert \frac{C ( x ) -\Gamma ( x+1 ) }{\Gamma ( x+1 ) } \vert $	$\vert \frac{W_{1} ( x ) -\Gamma ( x+1 ) }{\Gamma ( x+1 ) } \vert $	$\vert \frac{W_{2} ( x ) -\Gamma ( x+1 ) }{\Gamma ( x+1 ) } \vert $
1	1.114 × 10^−4^	1.398 × 10^−4^	1.832 × 10^−4^	2.407 × 10^−5^
2	1.900 × 10^−6^	2.222 × 10^−6^	2.668 × 10^−6^	2.308 × 10^−7^
5	4.353 × 10^−9^	4.956 × 10^−9^	5.743 × 10^−9^	1.249 × 10^−10^
10	3.609 × 10^−11^	4.088 × 10^−11^	4.710 × 10^−11^	2.785 × 10^−13^
20	2.864 × 10^−13^	3.240 × 10^−13^	3.727 × 10^−13^	5.634 × 10^−16^
50	4.713 × 10^−16^	5.330 × 10^−16^	6.129 × 10^−16^	1.492 × 10^−19^
100	3.684 × 10^−18^	4.166 × 10^−18^	4.791 × 10^−18^	2.918 × 10^−22^

## References

[1] Anderson G.D., Vamanamurthy M.K., Vuorinen M. (1997). Conformal Invariants, Inequalities, and Quasiconformal Maps.

[2] Anderson G.D., Vamanamurthy M.K., Vuorinen M. (2007). Topics in special functions II. Conform. Geom. Dyn..

[3] Wang M.K., Chu Y.M., Song Y.Q. (2016). Asymptotical formulas for Gaussian and generalized hypergeometric functions. Appl. Math. Comput..

[4] Wang, M.K., Li, Y.M., Chu, Y.M.: Inequalities and infinite product formula for Ramanujan generalized modular equation function. Ramanujan J. (2017). 10.1007/s11139-0176-9888-3

[5] Wang M.K., Chu Y.M., Jiang Y.P. (2016). Ramanujan’s cubic transformation inequalities for zero-balanced hypergeometric functions. Rocky Mt. J. Math..

[6] Wang M.K., Chu Y.M. (2017). Refinements of transformation inequalities for zero-balanced hypergeometric functions. Acta Math. Sci. Ser. B Engl. Ed..

[7] Burnside W. (1917). A rapidly convergent series for $\log N!$. Messenger Math..

[8] Gosper R.W. (1978). Decision procedure for indefinite hypergeometric summation. Proc. Natl. Acad. Sci. USA.

[9] Batir N. (2008). Sharp inequalities for factorial *n*. Proyecciones.

[10] Mortici C. (2010). On the generalized Stirling formula. Creative Math. Inform..

[11] Ramanujan S. (1988). The Lost Notebook and Other Unpublished Papers.

[12] Smith, W.D.: The gamma function revisited (2006). http://schule.bayernport.com/gamma/gamma05.pdf

[13] http://www.rskey.org/CMS/the-library/11

[14] Mortici C. (2015). A new fast asymptotic series for the gamma function. Ramanujan J..

[15] Nemes G. (2010). New asymptotic expansion for the gamma function. Arch. Math. (Basel).

[16] Yang Z.-H., Chu Y.-M. (2015). Asymptotic formulas for gamma function with applications. Appl. Math. Comput..

[17] Chen C.-P. (2016). A more accurate approximation for the gamma function. J. Number Theory.

[18] Batir N. (2008). Inequalities for the gamma function. Arch. Math..

[19] Mortici C. (2009). An ultimate extremely accurate formula for approximation of the factorial function. Arch. Math..

[20] Mortici C. (2010). New sharp inequalities for approximating the factorial function and the digamma functions. Miskolc Math. Notes.

[21] Mortici C. (2011). Improved asymptotic formulas for the gamma function. Comput. Math. Appl..

[22] Zhao J.-L., Guo B.-N., Qi F. (2012). A refinement of a double inequality for the gamma function. Publ. Math. (Debr.).

[23] Mortici C. (2013). Further improvements of some double inequalities for bounding the gamma function. Math. Comput. Model..

[24] Qi F. (2014). Integral representations and complete monotonicity related to the remainder of Burnside’s formula for the gamma function. J. Comput. Appl. Math..

[25] Lu D. (2014). A new sharp approximation for the gamma function related to Burnside’s formula. Ramanujan J..

[26] Lu D., Song L., Ma C. (2015). Some new asymptotic approximations of the gamma function based on Nemes’ formula, Ramanujan’s formula and Burnside’s formula. Appl. Math. Comput..

[27] Yang Z.-H., Tian J.F. (2017). Monotonicity and inequalities for the gamma function. J. Inequal. Appl..

[28] Alzer H. (1997). On some inequalities for the gamma and psi functions. Math. Comput..

[29] Yang Z.-H. (2016). Approximations for certain hyperbolic functions by partial sums of their Taylor series and completely monotonic functions related to gamma function. J. Math. Anal. Appl..

[30] Alzer H. (2009). Sharp upper and lower bounds for the gamma function. Proc. R. Soc. Edinb..

[31] Lu D., Song L., Ma C. (2014). A generated approximation of the gamma function related to Windschitl’s formula. J. Number Theory.

[32] Chen C.-P. (2014). Asymptotic expansions of the gamma function related to Windschitl’s formula. Appl. Math. Comput..

[33] Qi F., Cerone P., Dragomir S.S., Srivastava H.M. (2009). Alternative proofs for monotonic and logarithmically convex properties of one-parameter mean values. Appl. Math. Comput..

[34] Tian J.F., Ha M.H. (2017). Properties of generalized sharp Hölder’s inequalities. J. Math. Inequal..

[35] Tian J.F., Ha M.H. (2018). Properties and refinements of Aczél-type inequalities. J. Math. Inequal..

[36] Abramowttz M., Stegun I.A. (1972). Handbook of Mathematical Functions with Formulas, Graphs and Mathematical Tables.

[37] Yang Z.-H., Chu Y.-M., Tao X.-J. (2014). A double inequality for the trigamma function and its applications. Abstr. Appl. Anal..

[38] Yang Z.-H., Tian J. (2018). Monotonicity and sharp inequalities related to gamma function. J. Math. Inequal..

[39] Yang, Z.-H., Tian, J.: Convexity and monotonicity for the elliptic integrals of the first kind and applications. arXiv:1705.05703 [math.CA]

